# Long-term follow-up of portal vein thrombosis in an American Cocker Spaniel with lobular dissecting hepatitis: a case report

**DOI:** 10.1186/s12917-021-03017-2

**Published:** 2021-09-30

**Authors:** Yumi Sakamoto, Keita Sato, Chieko Ishikawa, Yumiko Kagawa, Tomohiro Nakayama, Manabu Sakai

**Affiliations:** 1 Department of Veterinary Medicine, College of Bioresource Sciences, Nihon University, 1866 Kameino, Fujisawa, 252-0880 Kanagawa Japan; 2 North Lab, 8–35 Kita, 2-chome Hondori, Shiroishi-ku, Sapporo, 003–0027 Hokkaido Japan

**Keywords:** Cocker spaniel, Computed tomography, Dalteparin, Dog, Portal hypertension, Portal vein thrombosis, Prednisolone

## Abstract

**Background:**

Lobular dissecting hepatitis (LDH) is a rare form of canine liver cirrhosis that may be accompanied by portal hypertension in American Cocker Spaniels. In human patients with liver cirrhosis, portal vein thrombosis (PVT) is a common complication. However, PVT has not been reported in dogs with LDH. Herein, we describe the long-term follow-up of PVT in an American Cocker Spaniel with LDH.

**Case presentation:**

An 8-year-old neutered male American Cocker Spaniel presented with a 1-month history of severe abdominal effusion. The dog was histopathologically diagnosed with LDH and treated with low-dose prednisolone on day 14. On day 115, computed tomography angiography (CTA) confirmed the presence of a thrombus in the portal vein. Therefore, the dog was subcutaneously administered with the anticoagulant dalteparin, and low-dose prednisolone was continued. As a follow-up for PVT, CTA examinations were performed on days 207, 515, 886, and 1168, and the dog’s antithrombin and D-dimer levels were measured. Following anticoagulant therapy, the dog was confirmed to have gradually increased antithrombin activity and decreased D-dimer concentrations. In addition, although the thrombus was confirmed to be in the same area of the portal vein system by CTA, atrophy and increased CT values due to organization were observed during the follow-up period. The dog’s condition remained stable without clinical signs until day 1112 when it developed hepatic encephalopathy. The dog died on day 1208. On postmortem examination, histopathologically, the liver showed marked bile duct hyperplasia and fibrosis with chronic thrombus in the portal vein.

**Conclusions:**

This case demonstrated that low-dose glucocorticoid combined with dalteparin allowed long-term follow-up of PVT in an American Cocker Spaniel with LDH.

## Background

American Cocker Spaniels are predisposed to chronic liver diseases, such as chronic hepatitis and liver cirrhosis; lobular dissecting hepatitis (LDH) also arises in this breed [1–3]. LDH is characterized by reticular fibers surrounding an individual or group of hepatocytes and is a form of liver cirrhosis according to World Small Animal Veterinary Association guidelines [4]. Dogs with LDH have shorter survival times than dogs with chronic hepatitis [5]. Intrahepatic portal hypertension (PH) is common in American Cocker Spaniels with chronic liver diseases [1, 2]. PH results in a high incidence of ascites and a formation of acquired portosystemic collaterals (APSCs) [1, 4, 6]. Hepatic encephalopathy is a sequela of APSCs. In particular, ascites is a negative prognostic indicator in dogs with chronic liver diseases [7]. However, a study reported that low-dose glucocorticoid therapy can prolong survival in American Cocker Spaniels who present with ascites due to severe chronic liver diseases, including LDH [1].

Portal vein thrombosis (PVT) is a rare thrombotic complication in dogs receiving glucocorticoid therapy and those with hepatic diseases, such as chronic hepatitis, hepatic neoplasia, and congenital portosystemic shunt [8, 9]. By contrast, PVT is common in humans with liver cirrhosis [10]. The thrombus increases the resistance of blood flow into the portal vein, which is a cause of prehepatic PH [11, 12]. Therefore, it is difficult to control ascites in liver cirrhosis patients with PVT.

Recently, computed tomography angiography (CTA) has been used to diagnose and follow up canine PVT [13–16]. Dogs with PVT have been treated with low molecular weight heparin, unfractionated heparin, clopidogrel, aspirin, and warfarin [8, 15]. However, to the best of our knowledge, there are no case reports of simultaneous treatment with glucocorticoid and anticoagulants in dogs with LDH and PVT. Herein, we describe the long-term follow-up of PVT in an American Cocker Spaniel with LDH.

## Case presentation

An 8-year-old neutered male American Cocker Spaniel was presented to the Nihon University Animal Medical Center with a 1-month history of severe abdominal effusion. The dog was treated with furosemide (2 mg/kg every 12 h orally), ursodeoxycholic acid (8 mg/kg every 12 h orally), and metronidazole (10 mg/kg every 12 h orally) for 1 month by the referring veterinarian. On first presentation, the dog was alert with a normal body condition. On physical examination, the dog presented with severe abdominal distention. Physical examination, including cardiovascular examination, was unremarkable. Moreover, complete blood count was unremarkable. A biochemical analysis of blood showed severe hypoalbuminemia (1.5 g/dL; reference range, 2.7–3.8 g/dL) and mildly increased activities of aspartate aminotransferase (73 U/L; reference range, 0–50 U/L) and gamma-glutamyl transferase (12 U/L; reference range, 0–7 U/L). Concentrations of serum bile acid were increased (fasting, 57 µmol/L; postprandial, 51 µmol/L; reference value, < 25 µmol/L), and blood coagulation screening tests revealed the following abnormalities: prothrombin time of 8.5 s (reference range, 6.0–8.0 s), activated partial thromboplastin time of 32.5 s (reference range, 10.0–16.0 s), and antithrombin activity of 67 % (reference range, 102–156 %). Abdominal radiographic examinations demonstrated microhepatica and loss of abdominal detail associated with abdominal effusion. Abdominal ultrasonography (US) revealed a large volume of free peritoneal fluid, an irregular liver surface, and hyperechogenicity in the hepatic parenchyma. Several small tortuous vessels were detected in the area of the left kidney using color flow Doppler imaging. Cytological analysis of peritoneal fluid was consistent with a low protein transudate. Bilirubinuria was detected on urinalysis. Based on these findings, the dog was suspected of having severe chronic liver disease. Therefore, food was changed to a liver-supportive diet, and the metronidazole dosage was decreased (7.5 mg/kg every 12 h orally). Furthermore, spironolactone was started (1 mg/kg every 12 h orally) in addition to the other previous medications prescribed by the referring veterinarian. Laparoscopic examinations, including hepatic sampling and CTA, were performed for a definitive diagnosis on day 3.

**Fig. 1:**
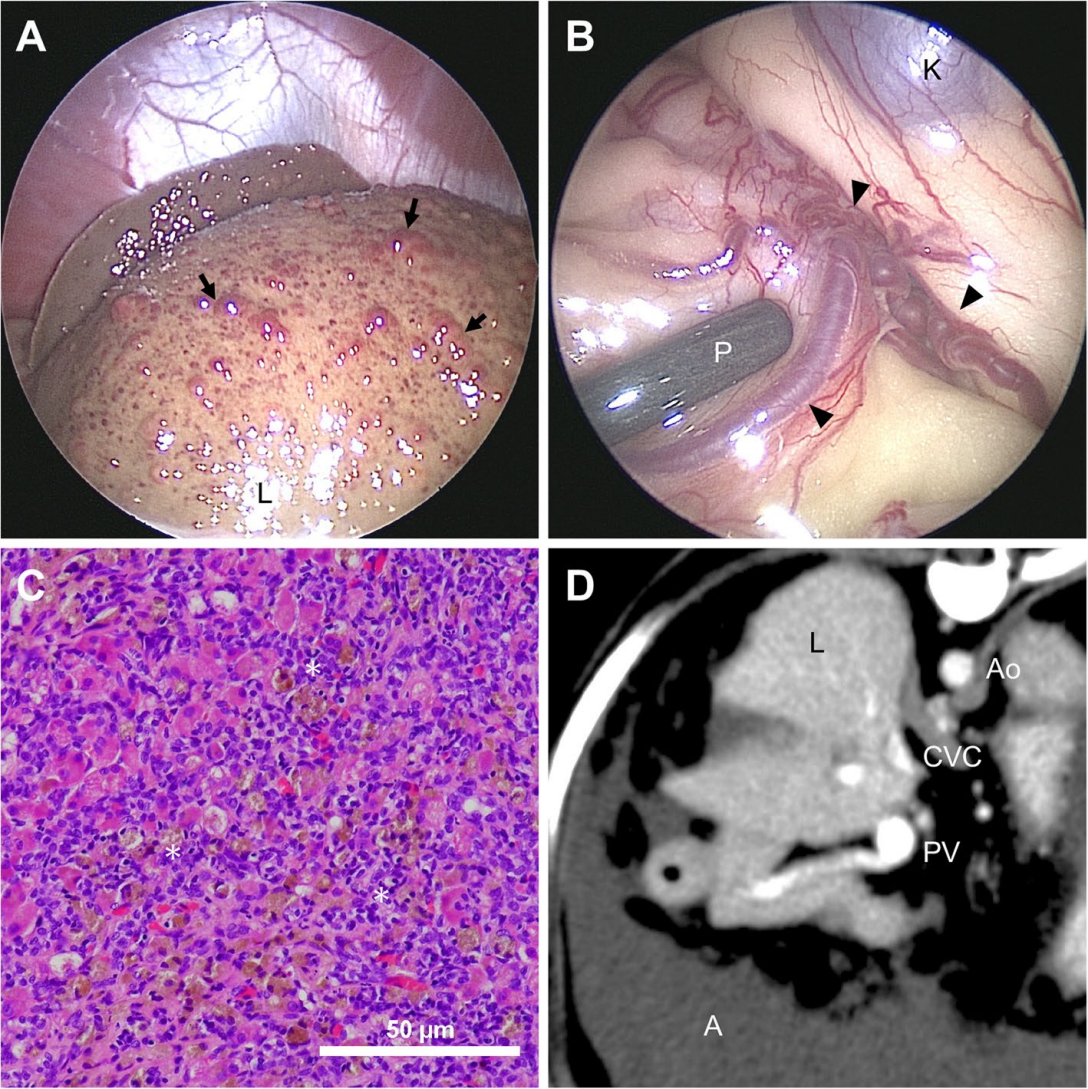
Laparoscopy, histopathology, and computed tomography angiography images from an American Cocker Spaniel on day 3. The micronodular formation (arrows) on the surface of atrophied left liver lobes (**A**) and the development of acquired portosystemic collaterals (arrow heads) around the area of the left kidney were observed using a 5-mm palpation probe (**B**). The normal liver architecture was disrupted by diffuse infiltration of hyperplastic bile ducts and inflammatory cells (asterisk) in the hepatic parenchyma, and some regenerative nodules were present on histopathology, hematoxylin and eosin stain (**C**). No evidence of a thrombus was found in the portal vein using computed tomographic angiography (**D**). A, ascites; Ao, aorta; CVC, caudal vena cava; K, kidney; L, liver; P, palpation probe; PV, portal vein

Laparoscopy revealed micronodular formations on the surface of the atrophied left liver lobes with multiple small regenerative nodules (Fig. 1A) and APSCs developing in proximity to the left kidney (Fig. 1B). Liver biopsy was performed using 5-mm laparoscopic biopsy forceps, and samples were obtained from both the wedge and surface of the left lobes. On histopathological examination (Fig. 1C), the normal liver architecture was disrupted by portal-portal bridging fibrosis (F4) and multiple regenerative nodules. Diffusely, the lobular structures had collapsed and were replaced by prominent hyperplastic small bile ducts and macrophages. Additionally, a few individual necrotic hepatocytes were observed. Furthermore, severe copper deposition was observed in periportal areas by rhodanine stain (Score 3), and the copper concentration was 1173.9 µg/g dry weight of the liver. No evidence of a thrombus was found in the portal venous system when analyzed using a 64-detector row computed tomography (CT) scanner (Aquilion 16, Canon Medical Systems, Tochigi, Japan) (Fig. 1D). Based on these results, the dog was diagnosed with severe LDH and intrahepatic PH. On day 14, the dog presented with a decreased severity of abdominal effusion. Therefore, furosemide administration was discontinued, and the dosage of spironolactone was increased to 2 mg/kg every 12 h orally. The dog was treated with prednisolone (0.25 mg/kg every 12 h orally) in addition to the previously prescribed medications, such as spironolactone (2 mg/kg every 12 h orally), ursodeoxycholic acid (8 mg/kg every 12 h orally), and metronidazole (7.5 mg/kg every 12 h orally). On day 28, the serum albumin concentration increased to 2.1 g/dL, and no evidence of ascites was detected on US. Therefore, the oral administration of prednisolone was tapered to a dose of 0.25 mg/kg every 24 h orally, and the dosage of spironolactone was decreased (1 mg/kg every 12 h orally).

**Fig. 2:**
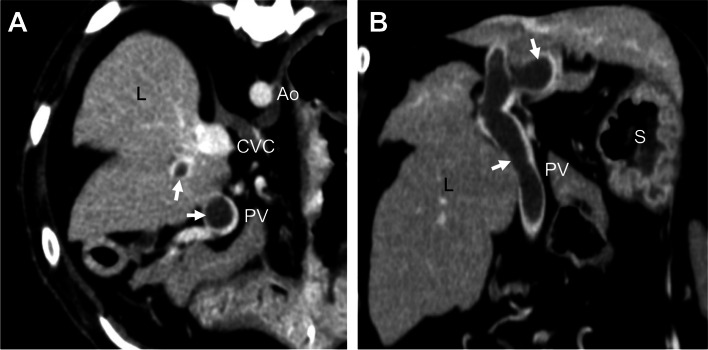
Portal vein thrombosis in an American Cocker Spaniel with lobular dissecting hepatitis on day 115. Transverse (**A**) and dorsal (**B**) computed tomography angiography images revealed a thrombus in the main portal vein, extending to the right and left branches of the intrahepatic portal vein (arrows). The thrombus in the dog was clearly detected as a non-contrast-enhanced structure, and the lumen of the blood vessel was enhanced. Ao, aorta; CVC, caudal vena cava; L, liver; PV, portal vein; S, stomach

On day 115, the dog arrived at our hospital for a regular recheck. The dog had a good general condition without abdominal distension. Laboratory findings revealed low antithrombin activity (66 %), mild thrombocytopenia (154,000/µL; reference range, 200,000 − 500,000/µL), and an increased D-dimer concentration (26.7 µg/mL; reference value, < 1.0 µg/mL). Abdominal US showed the presence of a 9.4-mm-diameter heteroechoic mass within the main portal vein at the level of the porta hepatis. Based on these findings, PVT was suspected. CTA was performed again, and the images revealed a thrombus in the main portal vein that extended to the right and left branches of the intrahepatic portal vein (Fig. 2). The medications were continued, such as ursodeoxycholic acid (8 mg/kg every 12 h orally), spironolactone (1 mg/kg every 12 h orally), and prednisolone (0.25 mg/kg every 24 h orally); anticoagulant therapy was started using dalteparin at a dose of 100 U/kg every 12 h subcutaneously.

**Fig. 3:**
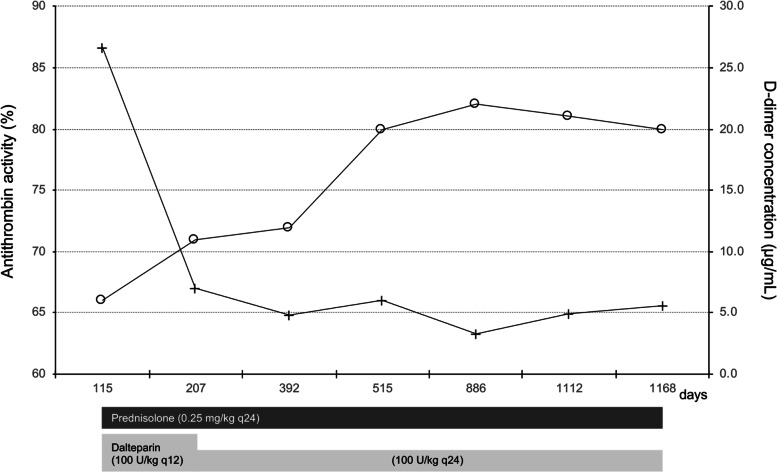
Time courses for changes in plasma antithrombin activity (circles) and D-dimer concentration (crosses) in an American Cocker Spaniel with lobular dissecting hepatitis and portal vein thrombosis treated with low-dose glucocorticoid combined with dalteparin

**Fig. 4:**
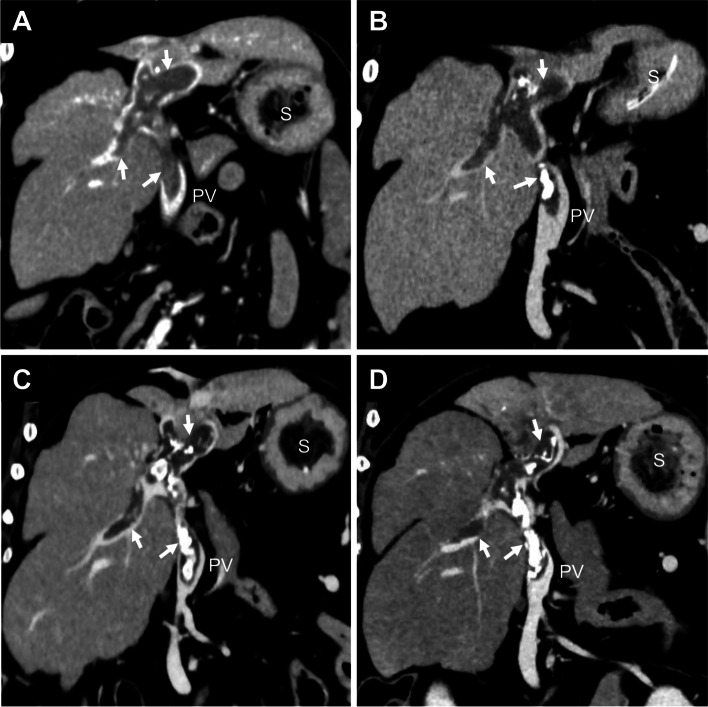
Dorsal computed tomography angiography images of the portal vein thrombosis (arrows) in an American Cocker Spaniel with lobular dissecting hepatitis on days 207 (**A**), 515 (**B**), 886 (**C**), and 1168 (**D**). The strong signal in the thrombus is an organized area. The size of the thrombus was slightly decreased, and the organized areas of the thrombosis were gradually increased. PV, portal vein; S, stomach

As a follow-up for PVT, plasma antithrombin and D-dimer levels were measured in the dog (Fig. 3), and CTA examinations were performed on days 207, 515, 886, and 1168 (Fig. 4). The dog’s condition remained stable without any clinical signs until day 1112. Therefore, the oral administration of spironolactone was tapered to a dose of 0.5 mg/kg every 24 h orally on day 207. After injections of dalteparin, the dog gradually showed increased antithrombin activity and decreased D-dimer concentrations, which did not return to the normal range throughout the treatment period. Additionally, albumin concentration remained at 2.0–2.1 g/dL during the follow-up period. The thrombus had a slight tendency to atrophy, with increased CT values due to organization. On day 1112, the dog presented with neurological symptoms, including head press and swirling motion, caused by hepatic encephalopathy without abdominal effusion. The fasting ammonia concentration indicated the upper limit of the reference value (98 µg/dL; reference range, 0–98 µg/dL). On day 1168, CTA showed no changes in the thrombus in the portal vein, and both spironolactone and ursodeoxycholic acid were discontinued. Hyperammonemia was difficult to control because the dog was anorexic and unable to consume lactulose and a liver-supportive diet. Eventually, the dog could no longer be treated with glucocorticoids and anticoagulants and died on day 1208.

**Fig. 5:**
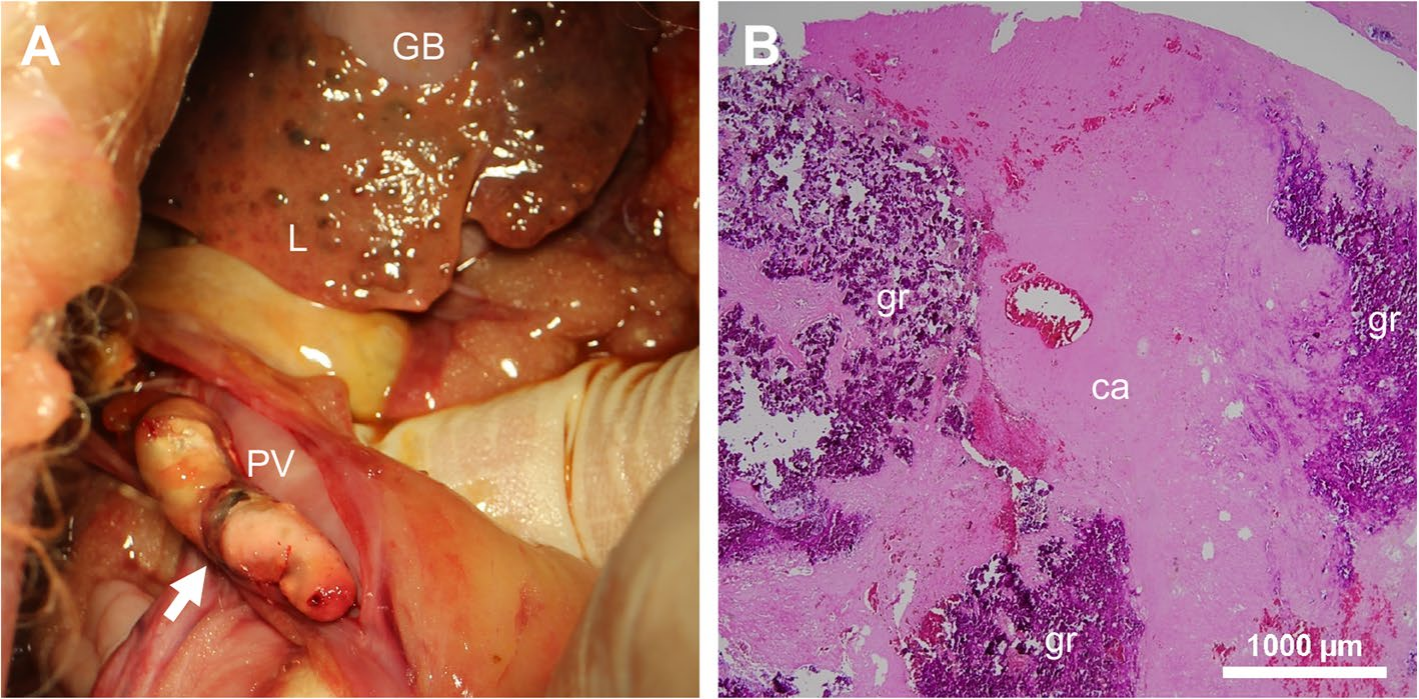
Autopsy findings in an American Cocker Spaniel with lobular dissecting hepatitis and portal vein thrombosis. The autopsy performed on day 1208 confirmed atrophied liver lobes with a number of small regenerative nodules and a large, hard thrombus (arrow) attached to the wall of the main portal vein (**A**). The thrombus showed calcification (ca) and granulation (gr) on histopathology, hematoxylin and eosin stain (**B**). GB, gall bladder; L, liver; PV, portal vein

Postmortem examination of the dog demonstrated atrophied liver lobes with multiple small regenerative nodules. In addition, a large (30 × 10 mm) thrombus was attached to the wall of the main portal vein (Fig. 5A). Histologically, a marked intralobular bridging fibrosis with proliferation of small bile ducts was noted. Furthermore, multifocal calcification and mild proliferation of granulation tissue were observed in the thrombus (Fig. 5B).

## Discussion and conclusions

LDH is a form of liver cirrhosis, which reflects the end stage of chronic hepatitis, and is histopathologically characterized by substantial architectural distortion, fibrosis, and intrahepatic sinusoidal PH [1, 2, 4]. An initial diagnosis of liver cirrhosis has been associated with a shortened survival time compared with that of chronic hepatitis in dogs [17]. Our patient presented with abdominal effusion and was diagnosed histopathologically with LDH, and laparoscopic examination confirmed the presence of APSCs. In dogs with chronic liver diseases, ascites is a negative prognostic indicator [7], and the development of APSCs is indicative of PH that leads to severe complications [12]. In a retrospective study, oral prednisolone treatment (1 mg/kg/day) was not beneficial for canine liver cirrhosis [17]. However, our patient was treated with low-dose prednisolone (0.25–0.5 mg/kg/day) and survived without ascites for more than 3 years. Our results agree with a report that showed American Cocker Spaniels with LDH who were treated with low-dose prednisolone had relatively long survival times [1]. Prednisolone may have suppressed inflammation and fibrosis in these dogs and supported liver function, such as albumin synthesis.

The prevalence of PVT in 33 dogs is relatively high (42 %) in 14 dogs with hepatic diseases such as chronic hepatitis [8]. In canine liver disorders, hypercoagulability associated with alterations in either primary or secondary hemostasis arises from increased procoagulant activity, decreased anticoagulant factor activity, altered vascular flow, and disordered fibrinolysis [18]. In humans, PVT is a fairly common complication of liver cirrhosis and may influence the prognosis of patients with this disease [10]. Hypercoagulability is caused by decreased levels of protein C, protein S, and antithrombin in human patients with liver cirrhosis [19, 20]. In our canine patient with LDH, antithrombin activity was remarkably low due to severe liver dysfunction. Antithrombin levels were decreased in healthy dogs treated with prednisone, which may lead to increased thromboembolic risk [21]. Furthermore, glucocorticoid therapy has been associated with PVT in dogs [8, 12, 22, 23]. Therefore, the administration of prednisolone may have partly triggered the formation of thrombosis in the portal vein of our patient. In addition, decreased velocity and stagnation of portal blood flow were shown to play a role in the development of PVT in human patients with cirrhosis [19]. In our dog, portal blood flow was stagnated by intrahepatic PH. The mechanism of hypercoagulability and pathogenesis of PVT are unclear in dogs with liver cirrhosis and LDH. However, we conclude that PVT in our case may have been associated with hypercoagulability due to low antithrombin activity, glucocorticoid therapy, and abnormalities of the portal vein system from intrahepatic PH.

Blood concentrations of D-dimer were elevated in cirrhotic human patients with PVT, and it was associated with the degree of liver dysfunction in these patients [24, 25]. In dogs, the D-dimer is a diagnostic tool for the early detection of thrombosis [26–28]; furthermore, an elevated D-dimer concentration has been reported in canine PVT [15]. In our canine patient with LDH, the D-dimer concentration was above normal on the day of PVT diagnosis, and the concentrations gradually decreased with the continuous injection of dalteparin. Thus, the D-dimer test was a useful monitoring tool for PVT over time in our dog with LDH. However, D-dimer concentrations in this dog continued to be above the normal range during treatment for LDH and PVT. In addition, corticosteroids have been shown to induce a state of hypercoagulability in healthy dogs, as demonstrated by abnormalities in thromboelastography parameters [29–31]. Therefore, D-dimer concentrations may have been affected by liver function and prednisolone therapy in our dog.

In humans with liver cirrhosis, CTA is the gold standard for the definitive diagnosis of PVT [32]. In a previous report of 33 dogs with PVT, thrombus formation in the portal vein was determined by abdominal US (27/33), necropsy (4/33), direct visualization at surgery (1/33), or CTA (1/33). Recently, however, CTA has been reported to be a good tool for detecting and monitoring canine PVT [13–16]. Additionally, CTA rapidly provides a considerable amount of information, including the presence of APSCs and the portal vein/aorta ratio in dogs with PH [15, 33]. In the present case, the thrombus was clearly detected in the portal vein system by CTA. Using CTA, changes in thrombus size and organization were followed in canines after treatment with dalteparin and rivaroxaban [15, 34]. However, no information is available regarding CTA images in canines diagnosed with LDH and PVT after long-term anticoagulant treatment.

As confirmed by postmortem examination, the administration of dalteparin in our patient resulted in a slight reduction in thrombus formation due to thrombus regression and gradual calcification during thrombus organization. The result of CTA suggested that dalteparin might be effective in our dog with PVT. However, heparin requires antithrombin to inactivate factor Xa and dalteparin must be injected subcutaneously in dogs [15]. Thus, dogs with low antithrombin activity due to LDH and liver cirrhosis may require alternative drugs, such as rivaroxaban. Rivaroxaban is a direct factor Xa inhibitor that can be administered orally.

In conclusion, the administration of low-dose glucocorticoid combined with dalteparin allowed long-term follow-up of LDH and PVT in an American Cocker Spaniel.

## Data Availability

The datasets and images used and/or analyzed during the current study available from the corresponding author on reasonable request.

## Abbreviations

APSCs: Acquired portosystemic collaterals
CT: Computed tomography
CTA: Computed tomography angiography
LDH: Lobular dissecting hepatitis
PH: Portal hypertension
PVT: Portal vein thrombosis
US: Ultrasonography

## References

[1] Kanemoto H, Sakai M, Sakamoto Y, Spee B, van den Ingh TS, Schotanus BA (2013). American Cocker Spaniel chronic hepatitis in Japan. J Vet Intern Med.

[2] Mizooku H, Kagawa Y, Matsuda K, Okamoto M, Taniyama H (2013). Histological and immunohistochemical evaluations of lobular dissecting hepatitis in American Cocker Spaniel dogs. J Vet Med Sci.

[3] Bexfield NH, Buxton RJ, Vicek TJ, Day MJ, Bailey SM, Haugland SP (2012). Breed, age and gender distribution of dogs with chronic hepatitis in the United Kingdom. Vet J.

[4] van den Ingh TSGAM, Van Winkle T, Cullen JM, Charles JA, Desmet VJ (2006). Morphological classification of parenchymal disorders of the canine and feline liver. WSAVA standards for clinical and histological diagnosis of canine and feline liver disease.

[5] Poldervaart JH, Favier RP, Penning LC, van den Ingh TS, Rothuizen J (2009). Primary hepatitis in dogs: a retrospective review (2002–2006). J Vet Intern Med.

[6] Webster CRL, Center SA, Cullen JM, Penninck DG, Richter KP, Twedt DC (2019). ACVIM consensus statement on the diagnosis and treatment of chronic hepatitis in dogs. J Vet Intern Med.

[7] Raffan E, McCallum A, Scase TJ, Watson PJ (2009). Ascites is a negative prognostic indicator in chronic hepatitis in dogs. J Vet Intern Med.

[8] Respess M, O’Toole TE, Taeymans O, Rogers CL, Johnston A, Webster CRL (2012). Portal vein thrombosis in 33 dogs: 1998–2011. J Vet Intern Med.

[9] Van Winkle TJ, Bruce E (1993). Thrombosis of the portal vein in eleven dogs. Vet Pathol.

[10] Qi X, Han G, Fan D (2014). Management of portal vein thrombosis in liver cirrhosis. Nat Rev Gastroenterol Hepatol.

[11] Bertolini G (2010). Acquired portal collateral circulation in the dog and cat. Vet Radiol Ultrasound.

[12] Buob S, Johnston AN, Webster CRL (2011). Portal hypertension: pathophysiology, diagnosis, and treatment. J Vet Intern Med.

[13] French JM, Twedt DC, Rao S, Marolf AJ (2019). Computed tomographic angiography and ultrasonography in the diagnosis and evaluation of acute pancreatitis in dogs. J Vet Intern Med.

[14] French JM, Twedt DC, Rao S, Marolf AJ (2020). CT angiographic changes in dogs with acute pancreatitis: a prospective longitudinal study. Vet Radiol Ultrasound.

[15] Sato K, Sakamoto Y, Sakai M, Ishikawa C, Nakazawa M, Cheng CJ (2020). Diagnostic utility of computed tomographic angiography in dogs with portal vein thrombosis. J Vet Med Sci.

[16] von Stade LE, Shropshire SB, Rao S, Twedt D, Marolf AJ (2021). Prevalence of portal vein thrombosis detected by computed tomography angiography in dogs. J Small Anim Pract.

[17] Favier RP, Poldervaart JH, van den Ingh TS, Penning LC, Rothuizen J (2013). A retrospective study of oral prednisolone treatment in canine chronic hepatitis. Vet Q.

[18] Kavanagh C, Shaw S, Webster CRL (2011). Coagulation in hepatobiliary disease. J Vet Emerg Crit Care (San Antonio).

[19] Qi X, Han G, He C, Yin Z, Zhang H, Wang J (2012). Transjugular intrahepatic portosystemic shunt may be superior to conservative therapy for variceal rebleeding in cirrhotic patients with non-tumoral portal vein thrombosis: a hypothesis. Med Sci Monit.

[20] Tripodi A, Primignani M, Chantarangkul V, Dell’era A, Clerici M, de Franchis RD (2009). An imbalance of pro- vs anti-coagulation factors in plasma from patients with cirrhosis. Gastroenterology.

[21] Romão FG, Campos EF, Mattoso CRS, Takahira RK (2013). Hemostatic profile and thromboembolic risk in healthy dogs treated with prednisone: a randomized controlled trial. BMC Vet Res.

[22] Díaz Espiñeira MM, Vink-Nooteboom M, Van den Ingh TS, Rothuizen J (1999). Thrombosis of the portal vein in a miniature schnauzer. J Small Anim Pract.

[23] Sinnott VB, Otto CM (2009). Use of thromboelastography in dogs with immune-mediated hemolytic anemia: 39 cases (2000–2008). J Vet Emerg Crit Care (San Antonio).

[24] Zhang DL, Hao JY, Yang N (2013). Value of D-dimer and protein S for diagnosis of portal vein thrombosis in patients with liver cirrhosis. J Int Med Res.

[25] Li Y, Qi X, Li H, Dai J, Deng H, Li J (2017). D-dimer level for predicting the in-hospital mortality in liver cirrhosis: a retrospective study. Exp Ther Med.

[26] Marschner CB, Kristensen AT, Rozanski EA, McEvoy FJ, Kühnel L, Taeymans O (2017). Diagnosis of canine pulmonary thromboembolism by computed tomography and mathematical modelling using haemostatic and inflammatory variables. Vet J.

[27] Nelson OL, Andreasen C (2003). The utility of plasma D-dimer to identify thromboembolic disease in dogs. J Vet Intern Med.

[28] Thawley VJ, Sánchez MD, Drobatz KJ, King LG (2016). Retrospective comparison of thromboelastography results to postmortem evidence of thrombosis in critically ill dogs: 39 cases (2005–2010). J Vet Emerg Crit Care (San Antonio).

[29] Rose LJ, Dunn ME, Allegret V, Bédard C (2011). Effect of prednisone administration on coagulation variables in healthy Beagle dogs. Vet Clin Pathol.

[30] Flint SK, Abrams-Ogg ACG, Kruth SA, Bersenas AM, Wood RD (2011). Independent and combined effects of prednisone and acetylsalicylic acid on thromboelastography variables in healthy dogs. Am J Vet Res.

[31] O’Kell AL, Grant DC, Panciera DL, Troy GC, Weinstein NM (2012). Effects of oral prednisone administration with or without ultralow-dose acetylsalicylic acid on coagulation parameters in healthy dogs. Am J Vet Res.

[32] Fujiyama S, Saitoh S, Kawamura Y, Sezaki H, Hosaka T, Akuta N (2017). Portal vein thrombosis in liver cirrhosis: incidence, management, and outcome. BMC Gastroenterol.

[33] Sakamoto Y, Sakai M, Watari T (2017). Portal vein/aorta ratio in dogs with acquired portosystemic collaterals. J Vet Intern Med.

[34] Sakamoto Y, Ishigaki K, Ishikawa C, Nakayama T, Asano K, Sakai M (2020). Successful management of portal vein thrombosis in a Yorkshire Terrier with protein-losing enteropathy. BMC Vet Res.

